# Innovation-Oriented Medical School Curricula: Review of the Literature

**DOI:** 10.7759/cureus.18498

**Published:** 2021-10-05

**Authors:** Jonathan Arias, Kyle W Scott, J.R. Zaldivar, Denslow A Trumbull, Blanka Sharma, Kyle Allen, Nikolaus Gravenstein

**Affiliations:** 1 Medicine, University of Florida College of Medicine, Gainesville, USA; 2 Neurosurgery, University of Florida College of Medicine, Gainesville, USA; 3 Anesthesiology, University of Florida College of Medicine, Gainesville, USA; 4 Surgery, University of Florida College of Medicine, Gainesville, USA; 5 Biomedical Engineering, University of Florida, Gainesville, USA

**Keywords:** surveys and questionnaires, medical education, leadership, entrepreneurship, curriculum

## Abstract

Innovation and entrepreneurship (I&E) programs in medical education have become available as medical schools recognize the need to train forward-thinking physicians. There is considerable diversity in the design and implementation of these curricula, which represents a challenge and possibly serves as a deterrent for the development of additional I&E programs. A comprehensive search of medical school I&E programs and review of all Association of American Medical Colleges member websites (n = 171) were conducted. This review sought to (1) identify all American and Canadian allopathic medical schools with I&E curricula, (2) evaluate their structure/integration in the context of medical education, (3) outline core learning themes, and (4) describe the evaluative metrics. Information was collected through published or publicly available websites and through a questionnaire sent to identified I&E program leaders. Twenty-eight I&E-oriented medical education programs were identified from 26 schools; all of the programs integrated faculty leadership with backgrounds in medicine, engineering, and/or business/entrepreneurship. Of the programs, 57% (16/28) had been launched within the past four years and 75% (21/28) based program enrollment on a selective application process. Nearly all (27/28) incorporated lecture series and/or hands-on modules as a teaching technique. The most prevalent metric was completion of a capstone project (22/28; 79%). At least 15.2% (26/171) of American and Canadian allopathic medical schools include the option for students to participate in an I&E curriculum-based program. This review can be used to help medical school faculty with developing I&E curricula.

## Introduction and background

The United States remains one of the most medically innovative countries in the world [1]. Within an industry as complex and dynamic as healthcare, medical school curricula should incorporate medical innovation training to proactively prepare students for the future. Following this principle, the American Medical Association includes systems-level problem solving as a requirement in medical education [2]. Similarly, innovation and entrepreneurship (I&E) skills are essential for physicians who want to impact their fields in a meaningful way [3]. These skills facilitate the recognition and analysis of a clinical or surgical challenge followed by the formulation of a design concept to effectively address the said challenge.

Historically, undergraduate medical education (UGME) has incorporated curriculum-based programs to enhance medical student mastery of different areas of focus such as entrepreneurship and public health [2,3]. In the past 13 years, a number of programs have been founded across multiple allopathic medical schools with one common objective, that is, to help bridge the gap between future physicians with an interest in developing I&E skills and engineers, entrepreneurs, and local innovators that can provide them with the resources to bring an innovative idea to fruition [2].

The specific goals and resources of I&E programs vary by school. Areas of program diversity include program structure, participant eligibility and selection process, core competencies, educational themes, teaching methods, and evaluative measures. Program structure ranges from dual-degree engineering programs to elective concentrations or curriculum tracks. Additionally, eligibility may be unrestricted or selective through an application process and a limited number of positions. Core competencies are program-specific goals that medical school administrators envision these students will achieve by completion. In this review, we identify the core competencies, as well as the teaching methods used in these programs, to identify common threads that may aid in developing I&E curricula nationwide.

To address the stated need for I&E programs complementary to UGME, our study hopes to aid their development by providing a framework for structure, core competencies, evaluative measures, and “best practices” of I&E-centered medical curricula. We conducted an exhaustive search of the published literature and websites of existing I&E programs, with an emphasis on answering the following three questions:

1. How are I&E programs organized and integrated with the medical school curriculum?

2. What are the core competencies of the I&E program?

3. How are the core competencies measured/evaluated?

We hope that our review can assist others seeking to implement similar I&E initiatives and thereby contribute to advancing I&E in healthcare.

## Review

Search of I&E programs

**Figure 1:**
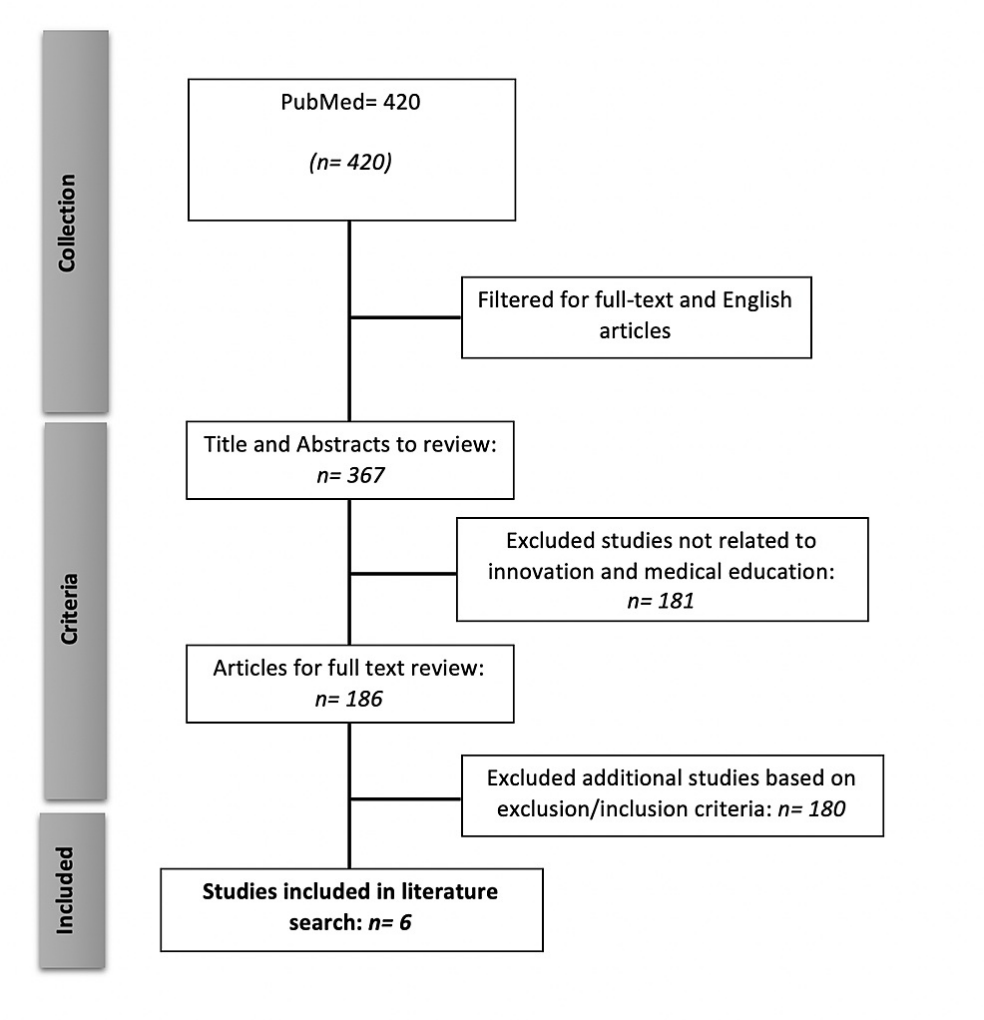
Flow diagram of study search methodology

Using PubMed, we conducted an exhaustive review of medical education curriculum development pertaining to innovation, entrepreneurship, and engineering. We performed this search on June 24, 2019, using the following search query: "Education, Medical"[Mesh] AND "Curriculum"[Mesh] AND ("Inventions"[Mesh] OR "Engineering"[Mesh] OR "Entrepreneurship"[Mesh]). From the 420 hits provided by this search, we excluded non-English articles and articles without full text. The remaining 367 articles were evaluated and excluded if they focused on undergraduate pre-medical curriculum, whereas innovation programs oriented toward medical school students were included. Studies directed toward residents, fellows, or non-medical school students and those focused on quality improvement or on the implementation of technology were also excluded. Additionally, we excluded studies that had no measurable outcomes, no structure, or no core competency data (e.g., surveys, reviews, observational studies). Reports involving a single case or the characterization of multiple cases of medical school I&E program development were included. After exclusion criteria were applied, six articles were left for analysis. Figure 1 shows a flow chart outlining the search process.

We also conducted a detailed review of all allopathic medical schools using the Association of American Medical Colleges member websites (n = 171) that include I&E components in their curriculum by 2019. We included programs that incorporate engineering, entrepreneurship, innovation, and/or business principles organized into an explicit and formal complementary course, track, or concentration in addition to the medical school curriculum. Schools that offer informal alternatives such as innovation hubs and offices of entrepreneurship devoid of any structure or curriculum goals and objectives were excluded. We collected information from each program through published or publicly available websites and downloadable material. Data collection was complemented through the administration of a focused questionnaire to the identified I&E program leaders to complete data points that were not easily or publicly accessible.

We sought to (1) identify and characterize all American and Canadian allopathic medical schools that incorporate I&E curricula into their UGME, (2) outline the most prevalent and overlapping teaching strategies and core competencies among all programs, (3) evaluate their structure and integration in the context of medical education, and (4) describe the evaluation metrics used.

Program design overview

We placed a specific emphasis on common curriculum design and components that demonstrate fulfillment of the I&E curriculum goals. Table 1 presents the most common teaching strategies (>50% of programs) in I&E curricula. Listed program strategies that use different nomenclature but similar themes were analyzed and synthesized into six core teaching themes through research team consensus. Specifically, the six strategies represent descriptive categories that group the following related approaches: (1) lecture series/seminars that include formal didactic or small-group discussion/conference, (2) progress meetings that include regularly scheduled check-ins to evaluate program advancement, (3) problem-based/team learning that includes practical and innovative application of I&E (e.g., capstone project), (4) workshops that include guided case-based problem solving, (5) mentorship that includes networking opportunities with faculty, and (6) guest lecturers that include outsourced lectures that are not typically part of the program.

**Table 1:** Common teaching strategies among innovation and entrepreneurship programs by medical school Note: Represented results were generated by grouping common themes into descriptive categories (six) and are non-comprehensive. ^a^Innovation and Entrepreneurship program in which marked category was optional.

Medical school	Teaching themes					
	Lecture series/seminars	Progress meetings	Problem/team-based learning	Workshops	Mentorship	Guest lecturers
Boston University School of Medicine	√				√	
Carle Illinois College of Medicine		√	√	√	√	
Columbia Vagelos College of Surgeons and Physicians	√		√^a^		√	
City University of New York (CUNY) School of Medicine	√		√		√	
Duke University School of Medicine	√		√		√	
Feinberg School of Medicine at Northwestern University	√		√	√	√	
George Washington School of Medicine and Health Sciences	√		√		√	
Harvard University Medical School	√		√	√	√	√
Icahn School of Medicine at Mount Sinai	√				√	
McGovern Medical School at UTHealth	√		√	√	√	
New York University School of Medicine	√		√	√	√	
Rutgers New Jersey Medical School	√	√	√		√	
Rutgers Robert Wood Johnson Medical School	√		√	√	√	
Texas A&M University College of Medicine	√		√		√	
Thomas Jefferson University Sidney Kimmel Medical College	√		√	√	√	
University of Arizona College of Medicine	√				√	
University of Florida College of Medicine	√		√		√	√
University of Illinois College of Medicine at Chicago	√		√	√	√	
University of Michigan Medical School	√		√		√	
University of Pennsylvania Perelman School of Medicine	√		√	√	√	
University of South Florida Morsani College of Medicine	√	√	√		√	
University of Southern California Keck School of Medicine	√		√	√	√	
University of Texas at Austin Dell Medical School	√		√		√	
University of Virginia School of Medicine	√		√	√	√	
Vanderbilt University School of Medicine	√		√		√	
Warren Alpert Medical School of Brown University	√	√	√		√	

The most ubiquitous educational subjects, which we termed core competencies, among identified I&E programs and found as a result of our literature search are listed in Tables 2, 3. We consolidated the most common core competencies (>50% of programs) into three core competencies representing a descriptive category that groups the following related themes: (1) law/regulation, but not limited to U.S. Food and Drug Administration regulation and healthcare regulation and policy, (2) business, but not limited to business finance and intellectual property, and (3) design/prototyping, but not limited to topics related to I&E plan formulation and implementation. This categorization facilitated the representation and standardization of the I&E programs we identified.

**Table 2:** Structure, core competency, and evaluation metrics among innovation and entrepreneurship programs by medical school Note: (1) Capstone represents a category that includes any scholarly project, scholarly concentration, thesis, manuscript, science projects, design projects, and/or grant proposals/investment pitches; (2) lectures and seminars were used as synonyms; (3) program structure, core competencies, and evaluation metrics are non-comprehensive. ^a^Summer option organized between years MS1 and MS2 and includes research, capstone-related projects, internships, and/or lectures/seminars. ^b^Competency represents a category that includes but is not limited to topics such as U.S. Food and Drug Administration regulations, patent law, and healthcare regulations and policy. ^c^Competency represents a category that includes but is not limited to topics such as business finance, business plan development, entrepreneurship overviews, and intellectual property. ^d^Includes additional (MS5) year in program (not labeled). MS, medical school

Medical school	Structure						Core competencies			Evaluation metrics
	MS1	MS2		MS3	MS4	Summer option^a^	Law/regulation^b^	Business^c^	Design/prototyping	
Boston University School of Medicine	Lectures							√		Grades
Carle Illinois College of Medicine				Clinical workshops; Capstone development					√	Capstone
Columbia Vagelos College of Surgeons and Physicians^d^	Lectures; capstone development						√		√	Capstone
City University of New York (CUNY) School of Medicine	Lectures; capstone development					√	√	√		Capstone
Duke University School of Medicine^d^				Lectures	Capstone development			√	√	Internship; capstone
Feinberg School of Medicine at Northwestern University	Lectures; shadowing	Workshops; case studies						√	√	Grades
George Washington School of Medicine and Health Sciences	Lectures			Capstone development		√		√		Attendance; evaluations; capstone
Harvard University Medical School	Single one-week intensive						√		√	Instructor Feedback
Icahn School of Medicine at Mount Sinai	Lectures						√	√		Grades
McGovern Medical School at UTHealth	Plan capstone	Lectures; workshops; capstone development			Finalize capstone	√			√	Capstone
New York University School of Medicine	Lectures	Extended summer research		Lectures; capstone		√	√			Lead journal club; capstone
Rutgers New Jersey Medical School	Lectures; plan summer project	Lectures; progress meetings; poster presentations			Lectures; meetings; capstone	√				Attendance; capstone
Rutgers Robert Wood Johnson Medical School	Lectures	Lectures; workshops		Capstone development		√	√	√	√	Attendance; grades; capstone
Texas A&M University College of Medicine	Lectures; capstone development							√	√	Grades; capstone
Thomas Jefferson University Sidney Kimmel Medical College	Lectures; workshops; case studies			Capstone development				√	√	Capstone
University of Arizona College of Medicine	Lectures							√	√	Grades
University of Florida College of Medicine	Lectures; guest lecturers		Capstone development			√	√	√	√	Attendance; capstone
University of Illinois College of Medicine at Chicago	Problem-based learning	Lectures; workshops		Capstone development		√		√	√	Grades; interview; capstone
University of Michigan Medical School	Lectures			Optional lectures	Capstone development			√	√	Grades; capstone
University of Pennsylvania Perelman School of Medicine	Lectures				Capstone development			√		Attendance; grades; capstone
University of South Florida Morsani College of Medicine	Lectures; progress meetings; capstone development					√	√	√		Hour minimum; capstone
University of Southern California Keck School of Medicine	Lectures; workshops; Capstone development						√	√	√	Attendance; grades; capstone
University of Texas at Austin Dell Medical School				Lectures; capstone development					√	Capstone
University of Virginia School of Medicine	Workshops				Lectures				√	Attendance
Vanderbilt University School of Medicine	Lectures	Capstone development			Lectures		√	√	√	Attendance; grades; capstone
Warren Alpert Medical School of Brown University	Plan capstone	Lectures; meetings		Capstone development		√		√	√	Capstone

**Table 3:** Structure, core competency, and evaluation metrics for innovation and entrepreneurship programs per literature review Note: Described program structure, core competencies, and evaluation metrics are non-comprehensive. ^a^Competency represents a category that includes but is not limited to topics such as U.S. Food and Drug Administration regulations, patent law, healthcare regulations and policy, and license agreements. ^b^Competency represents a category that includes but is not limited to topics such as business finance, business plan development, entrepreneurship overviews, intellectual property, economics, and health technology. ^c^Competency represents a category that includes but is not limited to topics such as Innovation and Entrepreneurship plan implementation.

Published literature	Structure	Core competencies			Evaluative metrics
		Law/regulation^a^	Business^b^	Design/prototyping^c^	
Cohen 2017 [4]	Weekly courses with problem-solving teams	√	√	√	Oral presentation
Gardner et al. 2018 [5]	Interdisciplinary problem-solving teams		√	√	Team competition
Niccum et al. 2017 [2]	Four-year curriculum	√	√	√	Evaluation
Servoss et al. 2018 [6]	One- to two-month course	√	√	√	Oral presentation
Stahlhut et al. 1997 [7]	One-month course		√		Oral presentation
Taylor et al. 2016 [8]	Five-year curriculum			√	Oral presentation

Program characteristics

A total of 28 I&E-oriented medical education programs from 26 institutions met the inclusion criteria out of the 171 (26/171; 15.2%) American and Canadian allopathic medical schools we examined (Table 4). Thomas Jefferson University Sidney Kimmel Medical College and University of Pennsylvania Perelman School of Medicine have two I&E programs each per inclusion criteria, which results in a total of 28 programs from 26 institutions. All of these programs integrate faculty leaders with backgrounds in medicine, engineering, and/or business/entrepreneurship. All programs award students upon successful completion of their parameters with at least one form of acknowledgment, most commonly a program-specific distinction on the student’s diploma and/or transcript or a stand-alone certificate during graduation.

**Table 4:** Characteristics of innovation and entrepreneurship programs at American and Canadian allopathic medical schools ^a^Schools that have more than one Innovation and Entrepreneurship program.

Medical school	Program name	Year founded	Program selectivity	Curriculum type	Dual degree	
						Boston University School of Medicine	Leadership and Innovation in Medicine and Business [9]	2015	Available to all medical students	Six-month course		
Carle Illinois College of Medicine	Innovation, Design, Engineering and Analysis (IDEA) Projects [10]	2018	Available to all medical students	One-year course		
Columbia Vagelos College of Surgeons and Physicians	MD-MS in Biomedical Engineering [11]	2018	Apply	Five-year dual-degree	√	
City University of New York (CUNY) School of Medicine	Master’s in Translational Medicine [12]	2015	Apply	One-year course	√	
Duke University School of Medicine	Doctor of Medicine-Master of Engineering Dual Degree (MD-MEng) [13]	2017	Apply	Five-year dual-degree	√	
Feinberg School of Medicine at Northwestern University	NUvention: Medical [14]	2007	Apply	Six-month concentration		
George Washington School of Medicine and Health Sciences	Clinical Practice Innovation and Entrepreneurship (CPI&E) Track [15]	2014	Apply	Four-year concentration		
Harvard University Medical School	MIT-HMS Healthcare Innovation Bootcamp [16]	2018	Apply	One-week course		
Icahn School of Medicine at Mount Sinai	Training in Biomedical Innovation & Entrepreneurship [17]	2017	Available to all medical students	Two-course concentration		
McGovern Medical School at UTHealth	Nanomedicine and BioMedical Engineering Scholarly Concentration [18]	2011	Apply	Four-year concentration		
New York University School of Medicine	Health Systems Innovation and Policy Concentration [19]	2012	Apply	Four-year concentration		
Rutgers New Jersey Medical School	Distinction Programs at Rutgers New Jersey Medical School [20]	2016	Apply	Four-year program		
Rutgers Robert Wood Johnson Medical School	Distinction in Medical Innovation and Entrepreneurship (DiMIE) [21]	2016	Apply	Four-year program		
Texas A&M University College of Medicine	Engineering Medicine (EnMed) [22]	2017	Apply	Four-year program	√	
Thomas Jefferson University Sidney Kimmel Medical College^a^	(1) Design (DES) Scholarly Inquiry Track; (2) Digital Health Track (DH) Scholarly Inquiry Track [23]	2012	Available to all medical students	Four-year track		
University of Arizona College of Medicine	Leadership and Innovation in Healthcare Distinction Track [24]	2009	Apply	Three- to four-year track		
University of Florida College of Medicine	Business and Innovation in Medicine Discovery Track [25]	2019	Available to all medical students	Four-year track		
University of Illinois College of Medicine at Chicago	Innovation Medicine Program [26]	2015	Apply	Six-week program		
University of Michigan Medical School	Innovation and Entrepreneurship Path of Excellence [27]	2015	Apply	>Two-year program		
University of Pennsylvania Perelman School of Medicine^a^	(1) Certificate in Healthcare Management, Entrepreneurship, and Technology (H-MET) [28]; (2) Commercialization and Entrepreneurship in Translation Program (CAET) [29]	2013	Apply	Four-year program		
University of South Florida Morsani College of Medicine	Innovation, Entrepreneurship and Business in Medicine Scholarly Concentration [30]	2007	Apply	Four-year concentration		
University of Southern California Keck School of Medicine	Health, Technology and Engineering (HTE@USC) Program [31]	2011	Apply	Four-year program		
University of Texas at Austin Dell Medical School	Design and Innovation Track [32]	2019	Apply	One-year track		
University of Virginia School of Medicine	UVA Medical Design Program [33]	2015	Available to all medical students	One-year program		
Vanderbilt University School of Medicine	Medical Innovators Development Program (MIDP) [34]	2018	Apply	Four-year PhD-to-MD program		
Warren Alpert Medical School of Brown University	Scholarly Concentration in Medical Technology, Innovation and Entrepreneurship [35]	2008	Apply	Four-year concentration		

I&E program characteristics, presented in Table 4, include year founded, selectivity, availability of a dual-degree option, and program length:

1. Year founded: The oldest I&E programs date back to 2007. Two programs were created and implemented as recently as the 2019 academic year. A total of 57% (16/28) of the identified programs were launched within the past four years.

2. Selectivity: Most programs (21/28; 75%) base enrollment on a selective application process after entry into the medical school. The application process typically consists of submission of a resume and personalized essay regarding the student’s motivation for pursuing the particular program.

3. Dual-degree option: Only four (14%) programs offer students the ability to earn a dual degree. Three of the four dual-degree programs focus heavily on the engineering aspect of medicine, with the other program being a one-year course focusing on translational medicine.

4. Program length: The most common (15/28; 54%) type of program is a four-year track/concentration, with variable organization and composition of responsibilities (Table 2). The remaining programs consist of a one-week concentration (n = 1), six-week course (n = 1), six-month concentration (n = 2), one-year course (n = 5), two-year program (n = 1), five-year program (n = 2), and a variable three- to four-year track (n = 1).

Core teaching strategies and themes

The following six common teaching strategies were identified (Table 1):

1. Lecture series/seminars (27/28; 96%): Nearly all programs use a formalized and logical succession of didactic sessions that emphasize their individual curricula educational objectives and goals. Didactic topics vary widely among programs, including U.S. Food and Drug Administration regulation, patent law, business-related topics, and prototyping (see Table 2 and/or the “Program Design Overview” section for more information).

2. Progress meetings (2/28; 7%): Interestingly, very few programs use designated conferences at predetermined regular intervals among team members to discuss milestone achievements and appropriate advancement.

3. Problem-based/team-based (25/28; 89%): A majority of programs incorporate the practical application of innovative skills in the form of identification and formulation of a capstone project with mentor guidance.

4. Workshops (13/28; 46%): Almost half of the identified programs use simulated experiences where students applied skill-based components of their I&E curricula. Notably, the Massachusetts Institute of Technology-Harvard Medical School Healthcare Innovation Bootcamp [16] from Harvard University Medical School heavily incorporates active learning experiences and practical application of innovation skills to simulated situations.

5. Mentorship (28/28; 100%): All programs heavily emphasize networking and guided learning with faculty.

6. Guest lecturers (2/28; 7%): Only two programs outsource qualified and renowned innovators and entrepreneurs from the local community to serve as guest lecturers. Both programs that incorporate this feature were founded within the last year. This is based on available data; therefore, some programs may have chosen not to publish this information.

Core educational competencies

We identified three educational competencies most frequently used among medical school I&E programs (Table 2). Business-oriented (20/28; 71%) educational competencies are most frequently incorporated into medical school I&E programs, with design/prototyping (19/28; 68%) and law/regulation (10/28; 36%) educational themes ranking second and third in prevalence, respectively. Only four (14%) of the 28 programs incorporate all three educational competencies, half (14/28; 50%) of the programs incorporate at least two of the educational competencies, and only one (1/28; 4%) program does not include any of our identified competencies.

Interestingly, our literature search on medical education curriculum development pertaining to innovation, entrepreneurship, and engineering revealed that the same three competencies are the most frequently used among medical school I&E programs (Table 3). Specifically, among the papers identified (n = 6), business-oriented (5/6; 83%) and design/prototyping (5/6; 83%) educational competencies are the most utilized, with law/regulation (3/6; 50%) being the least utilized.

Program structure

The integration of medical school I&E program components within the context of the core medical education timeline is outlined in Table 2. Generally, transitions in I&E program teaching themes parallel the progression of the core medical curricula at that particular institution [2]. Namely, the I&E lecture series/seminars, workshops, and guest lecturers are held during the medical program’s preclinical period, whereas capstone projects and progress meetings are commonly scheduled during the students’ clinical years. Specifically, capstone projects typically span the entire program length; however, capstone identification and planning occurs during the preclinical years, whereas actual capstone research and development occurs during the students’ clinical years. Additionally, lecture series/seminars are the teaching theme, with the greatest proportion taking place during the preclinical years when compared to other teaching modalities.

Several programs (10/28; 36%) integrate a summer experience related to the program’s objectives and goals and are exclusively allocated in the summer between the medical students’ first and second years. The summer experience varies significantly among programs including but not limited to capstone-related ventures, internships, and/or additional lecture series/seminars. Of the programs that offer a summer experience in their I&E program, the majority (8/10; 80%) are four-year programs, while the remaining two programs are a one-year (Master’s in Translational Medicine) program and a six-week (Innovation Medicine Program) program.

Program evaluative metrics

Our study identified several evaluative metrics that I&E programs use to gauge successful and satisfactory completion of its components (Table 2). The most prevalent metric is completion of a capstone project (22/28; 79%). Other examples include course grades, instructor evaluations, and attendance. Specific criteria constituting an eligible capstone project vary by program and have specific guidelines and requirements. Only half (14/28; 50%) of the programs include more than one evaluative metric.

Discussion

The upward trend in developing medical school I&E programs demonstrates both the appetite and student demand for training in physician-driven innovation. Moreover, there is increased collaboration and formalization of these programs across medical schools. Our search of both allopathic medical schools and the published literature on I&E programs identified trends and differences between these programs.

Niccum et al. [2] examined medical school curriculums and identified 13 I&E programs in 2016. They offered an overview of five program characteristics, common educational themes, and teaching methods used to instruct medical students with an interest in the field [2]. Their article is a significant contribution to the continuing discussion of I&E development among medical education and is a foundational component of this current review. We expounded on Niccum et al.’s publication and identified 28 I&E-oriented medical education programs in 26 schools. Most (57%) of these programs were launched within the past four years. In addition, 75% of the eligible curricula base their enrollment on a selective application process. The time commitments and initial student competency in innovation vary immensely between program goals and requirements. Some only admit students with technical backgrounds, whereas others require 14 hours of coursework or more before students are able to work on their capstone projects [4]. The most common type of I&E program is a four-year curriculum track/concentration.

The integration of I&E programs with medical school curricula remains one of the most challenging aspects of their development and implementation. On its own, medical school curricula and the educators who develop them face an enormous challenge due to the amount of material to cover. An additional facet of education such as I&E can strain students’ capacity for even more material and time commitment, making it difficult to incorporate it into an already crowded schedule. From our search, the majority (15/28; 54%) of programs span the full four years of medical school. Many programs that extend multiple years require the completion of a capstone project and typically only have informal meetings and discussions during the clinical phase of medical school education [2]. Thus, I&E programs of longer duration have the advantage of providing the time for more meaningful, long-term experiences. However, shorter programs may more easily fit within medical school curricula but lack the capacity for longitudinal experiences. Furthermore, we found that the majority of programs use lecture series/seminars (27/28; 96%), problem-based/team learning (25/28; 89%), and mentorship (28/28; 100%). These pedagogical approaches are expected as the skills being taught in I&E-type programs are divided among content knowledge, implementation, and experiences. Content knowledge can be efficiently delivered through lectures, whereas the implementation and experiences are best provided through mentorship and team-based learning. At many programs, the delivery of content is made flexible through the use of recorded lectures, online learning modules, take-home readings, flexible group meetings, and/or sessions with mentors. The necessity to deliver I&E material via multiple mediums presents a unique challenge to integrating their structure within the UGME framework, as the resources and administrative coordination of this material differ based on the delivery method. Thus, while many schools go to great lengths to plan I&E programs around various milestones and unavoidable time demands specific to each school, even the best prepared program may find it difficult to devote enough time to the meaningful achievement of its I&E program’s core competencies.

The core competencies and components that are present in the curriculum of a successful I&E program are highly variable by program. Moreover, a lack of standardization across programs is evident and typical for an emerging area of education. In an effort to quantify the core competencies, we divided curricular components into one of three categories: law/regulation, business, and design/prototyping. The lack of uniformity and breadth of topics implemented by existing I&E programs is likely a result of the enormous interdisciplinary scope inherent to most of them. After all, entrepreneurship encapsulates areas of engineering, business, and law, all topics to which one could easily devote years of study to master. Given the various starting levels of participants and the breadth of potential material to cover, most programs advocate for a broad covering of most topics, which gives students the necessary exposure to at least communicate effectively with specialists in each topic. With respect to content delivery, most programs advocate for interdisciplinary instruction and utilization of local departments/organizations for the delivery of the content. International studies into the optimal learning conditions of students in I&E programs also report the importance of implementing interdisciplinary professionals in the curriculum [36].

Adequate evaluation is essential to demonstrate and measure mastery of the core competencies of I&E programs. Given the breadth of experiences, variability in core competencies, and subjectivity of the content taught, measuring I&E program mastery remains a widely variable and challenging endeavor. We found that the vast majority (22/28; 79%) of medical school I&E programs implement a capstone project to evaluate content mastery (Table 2). All of the published I&E programs use some form of final project (e.g., oral presentation, team competitions, project evaluation) to determine successful mastery of I&E competencies (Table 3). These measures of competency parallel the unique challenges of I&E programs that need to ensure comprehension of fundamentals and lecture material (via quizzes, attendance, and grades) while also assessing student’s ability to implement these fundamentals and demonstrate the aptitude to pursue the innovative process (via capstones and final presentations). Some programs incorporate real-world components into their evaluation of team success through the adoption of experiences similar to “Shark Tank” or work with angel investors to create engaging challenges for the teams to solve [4]. Regardless of the medium used, any type of emerging I&E program will likely need to capitalize on both of these metrics and will have to depend on the experts of each core competency to ensure holistic and competent use of various interdisciplinary fundamentals in the innovation process.

Based on the responses to the survey data from medical I&E curricula program directors, we were able to identify key best practices that make for a successful I&E program. Many programs identified the use of industry partners and community entrepreneurs as providing perspective and a real-world context for the material presented in the classroom. Interdisciplinary teams and collaboration across departments were other crucial components identified by program directors. This allowed for the development of teams and capstone projects that extended across disciplines and most closely related with experiences encountered in industry. The third trend identified as a best practice across I&E programs was the adoption of a mentorship-oriented structure for help with student projects/capstones.

The search outlined in this study represents the largest and most inclusive compilation of medical school I&E programs to date. While the data collected for this study should aid in the portrayal of major trends and practices of allopathic medical school I&E programs, it is not without limitations. For instance, in spite of our efforts to gather and standardize as much public and program-specific data as possible, the results provided in this study are limited to the accuracy and extent of information obtained through websites and survey correspondences. Thus, the information included in this study could be subject to out-of-date websites or statements that may have been generalized incongruently across programs. Given that much of the data were found through a systematic web search of medical school webpages, it is possible that this search does not include I&E programs being implemented at medical schools that have little or no documentation on the web. This would cause our study to underestimate the actual prevalence of I&E programs and their practices. Additionally, our literature study found highly variable program development ranging from quality improvement oriented to those directed at residents or faculty [6,37,38]. Lastly, our study may also be limited in scope in that we assume all programs have the same goals and context rather than providing a framework for navigating the specific challenges and objectives for each program. As with the standardization of any highly heterogeneous dataset, our study is limited in that it serves mainly to demonstrate central themes and major practices from I&E programs inclusive of medical school students.

This study provides a framework for how existing programs are structured and integrated with medical school curricula, and how they measure the fulfillment of specified core competencies. We hope to both elucidate the practices being implemented at current programs and contribute to the conversation on how to best develop future I&E programs. It is important to note that these programs, while sharing some commonalities, will vary due to immense differences in specific goals, available institutional resources, and unique constraints. Thus, our study hopes to allow for the capitalization of best practices and promote transparency in the crucial components that allow for the growth of tomorrow's physician innovators. To this effect, we hope to see increased work done to promote collaboration on the development and formalization of these essential programs.

## Conclusions

As medicine continues to benefit from human ingenuity in its quest for a better understanding and treatment of disease, innovation-centered curricula have recently seen a dramatic increase in the UGME setting. Given the absence of a literature review on I&E curricula in UGME and the lack of standardization of “best practices” for these programs, this review’s objective was to gather the relevant information concerning the structure, core competencies, and evaluative measures used in American and Canadian allopathic medical schools. We observed a steep increase in the number of I&E programs since 2007, with most being founded within the past four years. The majority of these programs are selective four-year concentrations/tracks that include lecture series/seminars, problem-based/team learning, and mentorship as core teaching methods. They place an emphasis on law/regulation, business, and design/prototyping as core competencies and on the completion of a capstone project as an evaluative measure for the mastery of expected I&E skills. In the search for “best practices,” we identified the use of industry partners and community entrepreneurs, interdisciplinary teams and collaboration across departments, and the adoption of a mentorship-oriented structure for the development of student projects/capstones as effective ways to enhance the achievement of program goals. With this framework in mind, we hope that this study will guide UGME in developing new I&E curricula. Future studies should evaluate upcoming I&E programs and the impact that existing ones have had on the field.
